# A study of dynamic emoji emotional responses based on rhythms and motion effects

**DOI:** 10.3389/fpsyg.2023.1247595

**Published:** 2023-09-12

**Authors:** Danni Yang, Mei Wang, Yutong Ren, Xiaoyan Dong, Tian Yang

**Affiliations:** School of Mechanical Engineering, Sichuan University, Chengdu, China

**Keywords:** dynamic emoji, valence, arousal, motion effect, rhythm

## Abstract

Dynamic emojis are a form of nonverbal communication used in social programs to express emotions during conversations. Studies have shown that different dynamic effects can influence users’ emotional perceptions. Previous studies have focused on the emotional responses elicited by static emojis, while the emotional responses to dynamic emojis have not been thoroughly explored. In this study, we examined the impact of 128 different dynamic effects, categorized into emotional types (HAHV, LAHV, HALV, and LALV), on users’ arousal and valence, and conducted semi-structured interviews to identify users’ preferred dynamic effects. The results revealed significant and positive correlations between the arousal levels of all dynamic emojis and the effects of rhythms. However, the impact of rhythms on the valence of dynamic emojis varied depending on the emotion types of emojis. Specifically, the effects of motion on the valence of dynamic high-valence emojis were found to be significant, whereas they were not significant for dynamic low-valence emojis. Based on these findings, we recommend considering following factors in the design of dynamic emojis, including rhythms, motion effects, motion range, emotional metaphors, and the creation of contrast.

## 1. Introduction

With the rapid development of computers, people began to rely more on smartphone instant messaging for social interaction. In instant messaging, people began to use emoticons to express their nonverbal emotions (Lo, 2008; Ganster et al., 2012). With the popularity of emoticons, many mainstream social applications have started to provide dynamic emoticons as a part of the chat, such as Wechat, Telegram, and Twitter. Compared with static emoticons, dynamic emoticons are considered to be more expressive and have stronger emotional resonance (An et al., 2022).

The most dominant emoticons in social apps are emojis, which are essentially emoticons that support character input. The appearance of emoji can be designed by different platforms, but the basic elements are similar. Emojis can be utilized as paralinguistic cues to express emotional meaning (Aldunate and González-Ibáñez, 2017), help understand messages (Tang and Hew, 2018), allow recipients to more correctly understand the level and direction of emotional expression (Lo, 2008), facilitate social interaction and interpersonal emotional expression, reduce negative emotions (Derks et al., 2008), and also add and complement richer emotional meaning and situational context (Rezabek and Cochenour, 1998; Cramer et al., 2016), convey some indirect meaning (Holtgraves and Robinson, 2020), and contribute to a larger social presence (Aldunate and González-Ibáñez, 2017).

Static emojis can no longer fully meet people’s various expression needs. Emoji Sentiment Analysis List (2023) in the Emojiall platform categorizes the emotional expression of emojis, and also shows the effect of emojis on different platforms, in which QQ, Wechat, Telegram, JoyPixel, Skype, etc. are designed with dynamic emoji effects. Wechat and Tencent QQ have also created their own dynamic effects for emoticons. These dynamic emojis are often presented in the form of GIF, MP4, or JSON. Mainstream social applications show that the diversity of emojis is evolving from a static state to a dynamic state. Emojis with dynamic effects can add vividness and immersion, and participants prefer richer forms of dynamic emojis. Zheng et al. (2022) proposed dynamic preferences based on user contextual semantics and sentiment similarity recommendations and supported users to design personalized emojis. Sentiment study of animated GIFs of Jiang et al. (2017) suggested that images or emojis with animated effects may be a more nuanced form of nonverbal communication than static emojis. However, participants’ affective tendencies and usage propensity for dynamic emojis remain unknown, and we further focused on the emotional responses to dynamic effects.

Alshenqeeti (2016) proposed that emojis belonged to a form of visual communication and that emojis were used as non-verbal cues to illustrate the intention and emotion behind context. Researchers have done many explorations on the emotional aspects of emojis (Cherbonnier and Michinov, 2022). The main approaches to emotional research are differential emotion theory and dimensional emotion theory (Salminen et al., 2008). Differential emotion theory treats emotions as distinct categories (e.g., happy, sad, angry, surprised, etc.), whereas dimensional emotion theory maps emotion as a combination of two or more dimensions, such as the affective space proposed by Bradley and Lang (1994), where each emotion can be represented by a bipolar emotional adjective of different dimensions, the most commonly used of which are valence and arousal (Gunes and Pantic, 2010). Valence refers to the participant’s level of pleasure, and arousal refers to the participant’s level of excitement. These two dimensions are also often applied to the assessment of emotional responses to emojis.

Schouteten et al. (2022) designed the valence × arousal circumplex-inspired emotion questionnaire (CEQ) to study the evaluation of food stimuli. Toet et al. (2018) proposed the EmojiGrid graphical self-report tool that measures food-related valence and arousal. Susindar et al. (2022) proposed the Iconic Communication of Emotions (ICE) for emotional state assessment. These studies proposed that emojis could be used as a tool for emotion assessment, further illustrating the accuracy and importance of emojis in emotional expression.

The following studies have focused more on the significance of emoji emotions themselves. Rodrigues et al. (2018) investigated seven affective dimensions of 153 emojis, including valence and arousal, resulting in a LEED database with normative numeric ratings. Jaeger et al. (2019) explored consumer interpretations of emojis, including 33 emojis that are common in emotional expression, and similarly evaluated their valence and arousal. Kutsuzawa et al. (2022) found U-shaped curves for the valence and arousal ratings of emojis and categorized emojis into a more nuanced set of six different clusters. Notably, there were some differences in the rating scores between these studies. Brito et al. (2019) explored the specific emotions conveyed by emojis, correlating positive, neutral, and negative valence with similar emotions of emojis, and found that only neutral emojis matched the neutral sentiment of written messages. Aldunate et al. (2018) investigated the interaction between emoji valence and content valence and found that negative emotions were perceived higher for positive emoji with inconsistent content and emoji emotions. Fischer and Herbert (2021) examined participants’ emotional judgments of emojis, emoticons, and human faces, and then asked participants to rate the affective intensity, arousal, and valence of three stimulus categories and six discrete emotions. The result showed that emojis elicited the highest arousal, and of these, the highest valence was for positive emotions related to happiness. The research on the emotion-related aspects of emojis has yielded some interesting results so far and has provided some insights into the role of emojis in emotional communication.

Several exploratory studies have been done previously on the emotions of dynamic images in computer communication, TV and movie interfaces, and the findings of these studies can provide ideas for the exploration of dynamic emojis. Detenber et al. (1998) examined how image motion affected viewers’ emotional reactions to images. Participants viewed dynamic and static versions of different images selected from movies and TV shows. The results showed that image motion significantly increased arousal, and people paid more attention to highly arousing images. Dynamic positive images were more positive compared with static positive images, and dynamic negative images were more negative. Simons et al. (1999) also found that motion increased arousal and maintained subjects’ attention to images, but no clear effect on valence was reported.

In dynamic effects, motion is the most basic and important part, and the most important part of motion is rhythm and time, and the time of motion directly affects the change of rhythm (Thalmann, 1990). Rhythm changes over time and is an expression of motion time. In animation or advertisement, rhythm in dynamic effects can be used to enhance the emotional experience and involvement of participants, and often to create tension or trigger emotional resonance. A study shows the effects of motion speed on physiology and psychology in online animated advertisements (Sundar and Kalyanaraman, 2004). They set fast-paced and slow-paced animations, and then measured participants’ arousal. The results showed that fast-paced animations were more attention-grabbing and had higher physiological arousal, while slow-paced animations seemed to enhance the overall attractiveness of the website. There is also a relationship between dynamic images and emotional factors in in-vehicle information interfaces (Kim et al., 2020). They explored three motor attributes: duration, easing type, and time interval, and asked participants to assess 12 stimuli using 11 bipolar emotional adjectives. The study found that “fluid” and “energetic” emotions were evoked by dynamic images, and “energetic” emotions were stronger for short intervals. These studies showed that rhythms were an important factor in the dynamic effect and had a moderating effect on emotion.

Previous authors have also explored the role of different motion effects in the emotional experience. Certain motion patterns had more emotional expression than others, and the researchers explored which motion structures elicited similar emotional perceptions in participants (Rimé et al., 1985). The results indicated that motion with simple scenarios was more likely to elicit emotional perceptions in observers, and the type of emotion elicited varied depending on motion types. Podevin et al. (2012) investigated the effect of basic motion types on participants’ emotional responses during cognition. Parabolic motion enhanced memory for negative words, while wave motion enhanced memory for positive words. Moreover, positive words were better remembered. The results suggested that the perception of basic motion triggered emotional responses during cognition and that emotional responses depended on the perceived types of basic motion. Chafi et al. (2012) further investigated the effect of three different motion patterns on the emotional evaluation of emotional faces based on Podevin et al. (2012). The results showed that the role played by motion effects in arousal was significant. Wave motion increased the perceived intensity of positive emotions such as “surprise” and “happiness.” Parabolic motion increased the perceived intensity of “sad” emotions, and translational motion had equal effects on the perceived intensity of positive and negative emotions. In terms of vocabulary memory and face emotion, dynamic motion effects play a certain role in emotional experience. In interface design, dynamic motion effects are also often used to cue participants about the current operation. Harrison et al. (2011) defined a motion-based image scheme, Kinecticon that could be applied to the manipulation of the Graphical User Interface. Kinecticon is a dynamic behavior that does not change the visual content of an element or the RGBA value of a pixel. This allows a set of kinetic behaviors to be used for a variety of purposes and also convey different messages and emotions. These studies suggested that different motion effects had an affective modulation role in cognitive processes, face emotion judgments, and interface design.

## 2. Purpose of the study

Currently, there have been numerous studies on the emotional response and recognition of static emoticons, but there is a lack of research focusing on the emotional response of emoticons with dynamic effects. This study aimed to investigate the impact of rhythms and motion effects on different emotion types of dynamic emojis. We measured arousal and valence in the affective space and conducted emoji evaluation experiments with dynamic effects. Additionally, we conducted semi-structured interviews during the experiments to gather participants’ opinions on each dynamic effect and their favorite dynamic emojis. Finally, based on the design of dynamic effects, we proposed suggestions for the design of dynamic emojis with different emotion types, providing a foundation for the development of dynamic emojis.

## 3. Materials and methods

### 3.1. Dynamic emojis design

**Figure 1:**
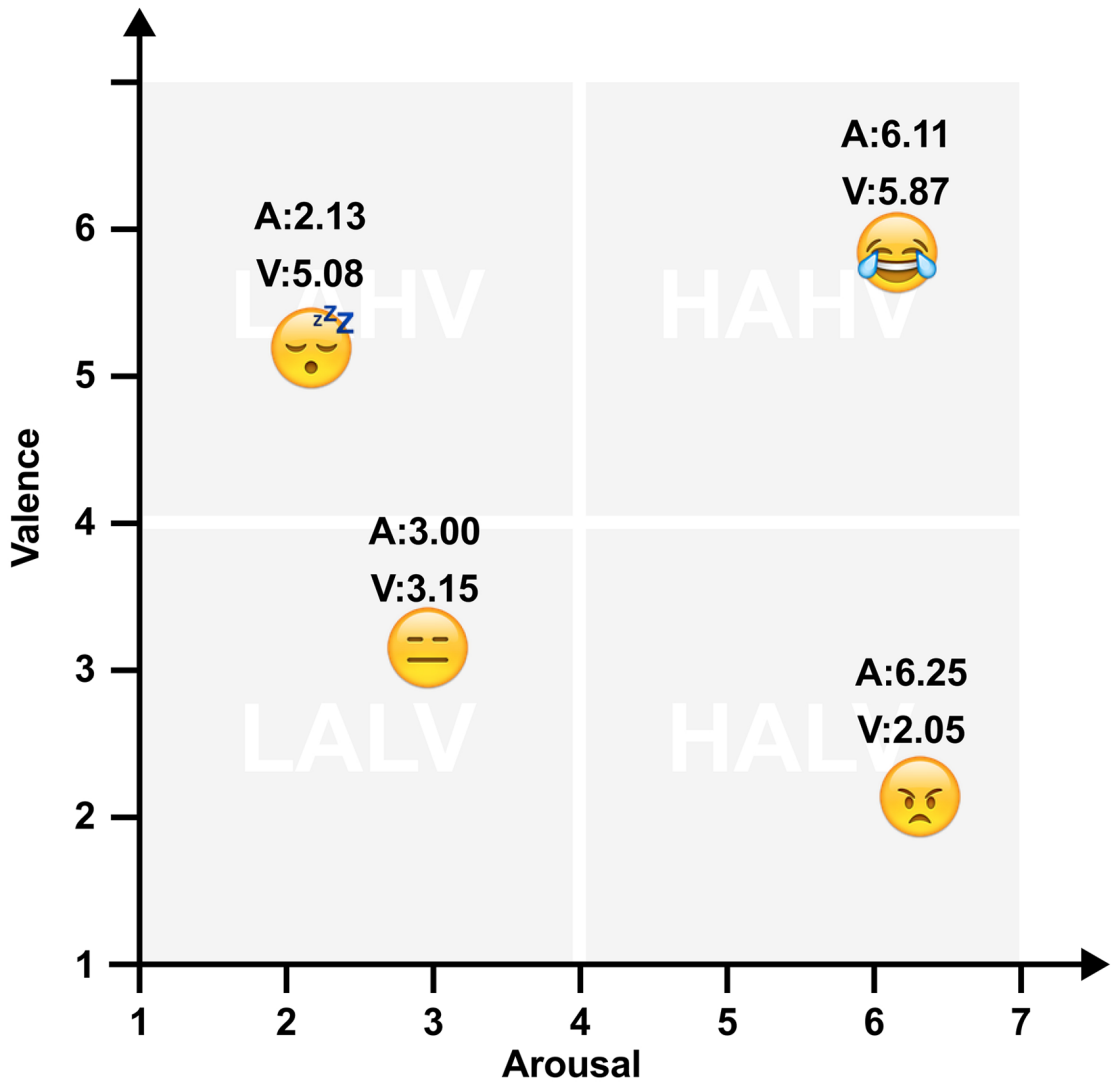
The four emojis were distributed in four quadrants of the affective Valence × Arousal space. Emojis reproduced with permission from the Unicode Foundation (http://unicode.org/emoji/charts/full-emoji-list.html).

The base ratings for static emojis referred to the LEED database (Rodrigues et al., 2018), as they included emojis for the largest number of operating systems (iOS, Android) and took into account the matching of emojis across operating systems with canonical systematic ratings, which could be used in our experiments to control for stimulus features. Taking into account possible differences between cultures and subjects, in our previous pre-experiment, we investigated user’s ratings for emojis with similar ratings in the database, which were often used and are not offensive. Finally, we chose as emoji material four iOS facial emojis distributed in the four quadrants of the emotional space in the LEED database. Their valence and arousal scores were shown in Figure 1 (ranging from 1 to 7). The representation and abbreviations of the four quadrants referred to the study of Koelstra et al. (2012), High-arousal and high-valence (HAHV), e.g., Tears of Joy (A: 6.11, V: 5.87). Low-arousal and low-valence (LALV), e.g., Expressionless (A: 3.00, V: 3.15). High-arousal and low-valence (HALV), e.g., Angry (A: 6.25, V: 2.05). Low-arousal and high-valence (LAHV), e.g., Sleeping (A: 2.13, V: 5.08).

**Figure 2:**
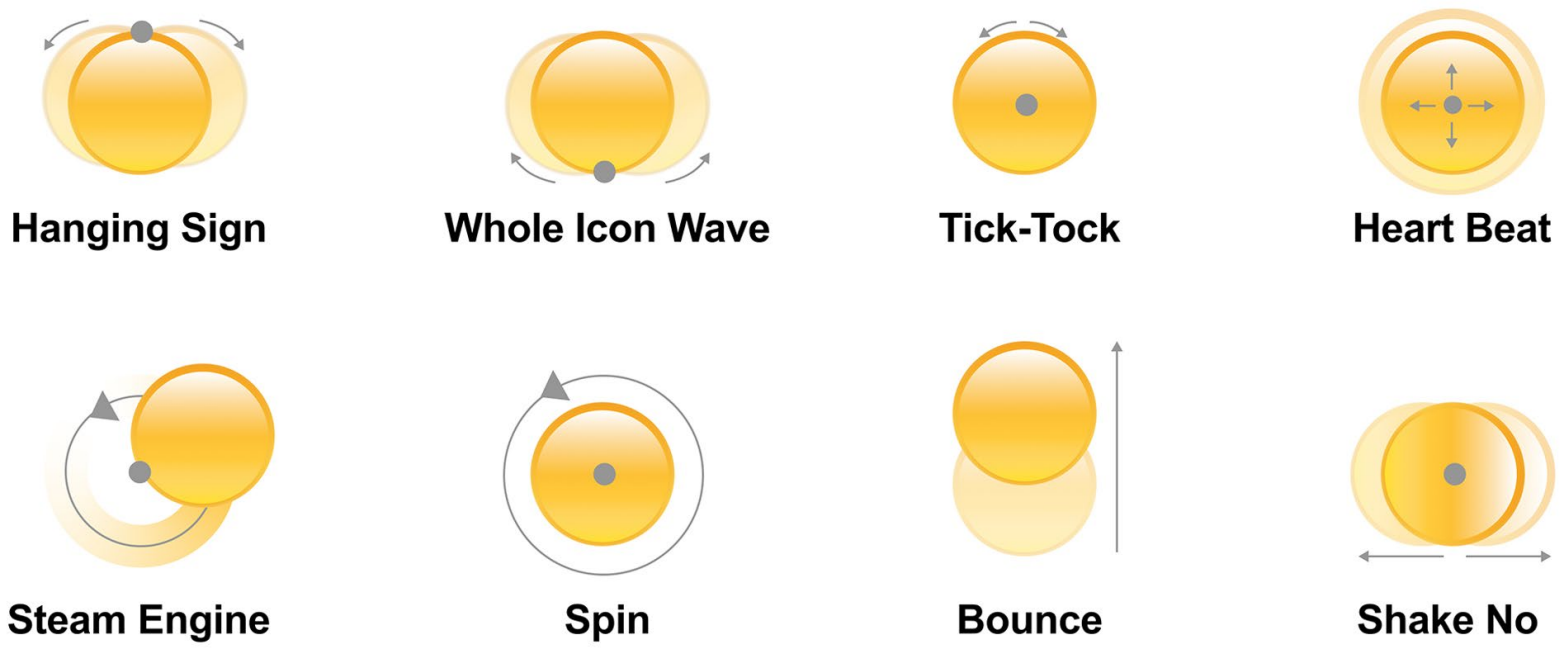
Schematic diagram of eight different motion effects. The gray dot represents the center of the motion, and the arrow indicates the direction of the emoji’s motion.

**Figure 3:**
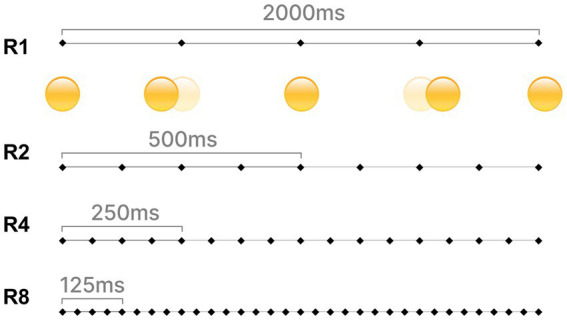
Each motion effect contains five key frames, and below R1 shows the motion effect of Bounce-R1. R1: Repeat motion effect once; R2: repeat motion effect twice; R4: repeat motion effect four times; and R8: repeat motion effect eight times.

Combining the motion effects of existing dynamic emoticons, eight types of motion that do not produce deformation in kinetic animations were selected for exploration (Harrison et al., 2011), where animations with similar motion paths were not repeatedly selected. The motion effects we selected include Hanging Sign (HS), Whole Icon Wave (WIW), Tick-Tock (TT), Heart Beat (HB), Steam Engine (SE), Spin (S), Bounce (B), and Shake No (SN), as shown in Figure 2. Hanging Sign and Whole Icon Wave have a 30-degree left/right oscillation angle. Tick-Tock has a 15-degree *in situ* oscillation angle. Heart Beat has a 1.2× magnification. Steam Engine and Spin have a counterclockwise rotation. Bounce and Shake No have a 1.5× stroke. The duration of the motion was chosen to be 2 s. We set four rhythms, which are the slowest rhythm (R1), the slower rhythm (R2), the faster rhythm (R4), and the fastest rhythm (R8), as shown in Figure 3. Finally we used After Effect to create dynamic effects for each of the four emojis, resulting in our final set of 128 dynamic emojis.

### 3.2. Participants

A total of 20 participants (10 males, 10 females) were recruited for the experiment. Ages of the participants ranged from 21 to 30. Every participant regularly used a smartphone and had no documented sensorimotor disabilities. After the trial, they were compensated appropriately.

### 3.3. Apparatus

The experiment used a smartphone (an iPhone 12 with a 6.1-in touchscreen) with a resolution of 1,170px × 2,532px, and dynamic emojis were displayed sequentially in the center of the phone screen, where participants could tap to play to see dynamic emojis.

### 3.4. Experiments design

**Figure 4:**
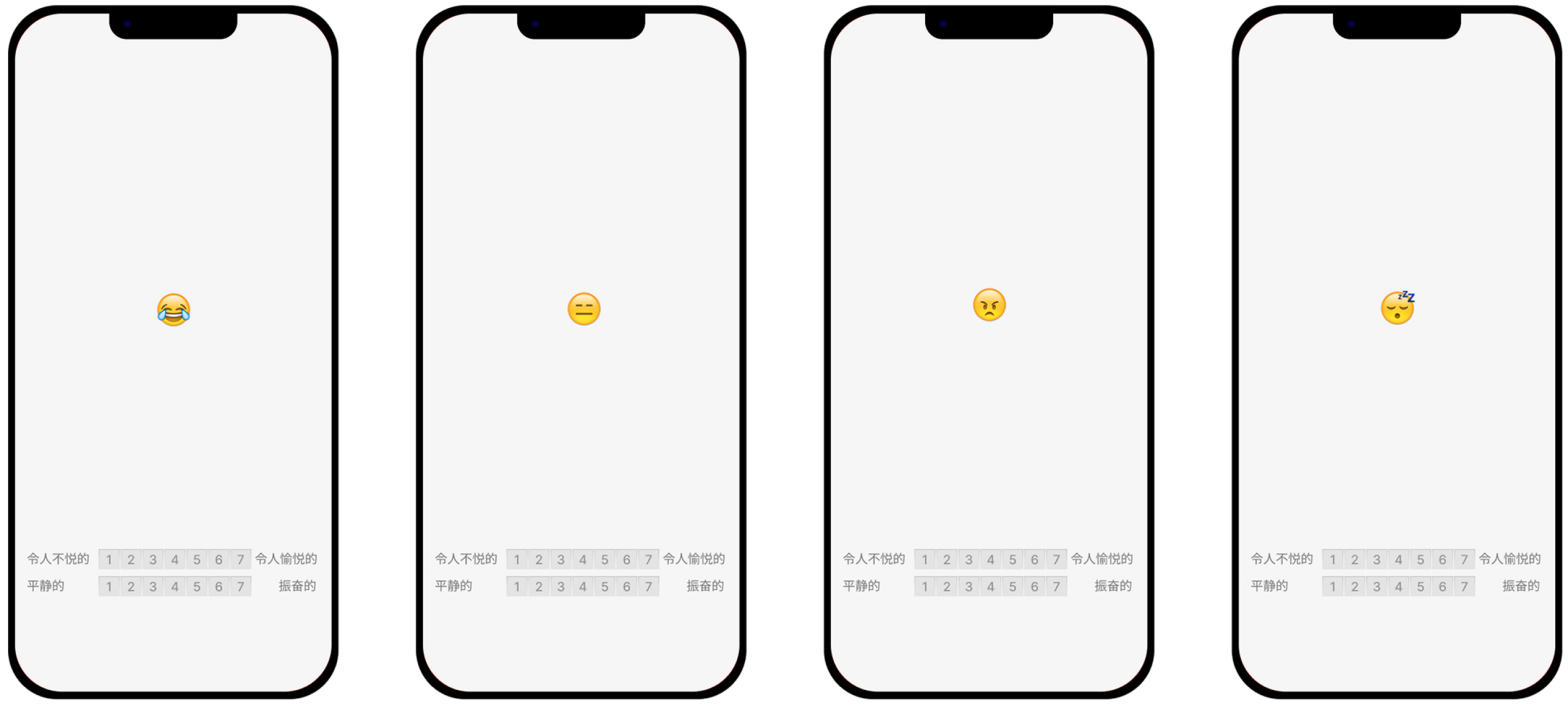
Video of the test with four emojis, with the emoji placed in the middle and the valence and arousal evaluation scales at the bottom. Emojis reproduced with permission from the Unicode Foundation (http://unicode.org/emoji/charts/full-emoji-list.html).

Every dynamic emoji was played in the video format with a frame rate of 60 fps and an emoji size of 80px × 80px, as shown in Figure 4. The experiments were conducted using a mixture of within-subject and between-subject design experimental methods, including four emojis, and the stimuli were divided into eight groups (HS, WIW, TT, SE, S, B, SN, and HB) according to the motion effects, and each group included four different animation rhythms (R1, R2, R4, and R8). For the four emojis, this was an 8 × 4 mixed design experiment with a total of 128 stimuli.

### 3.5. Procedure

The entire experiment was divided into two parts: a subjective questionnaire and a semi-structured interview. Participants were seated in chairs in an empty room, and the experimenter explained the concepts of valence and arousal. Participants were informed that the main purpose of the experiment was to rate the valence and arousal elicited by each dynamic emoji on a Likert scale (1–7). Participants were free to express their opinions at any time during the experiment, and a semi-structured interview was held at the end of the experiment. Before the formal experiment, we randomly selected 12 dynamic effects from four emojis as a training group to help users familiarize themselves with the testing process and establish their own rating criteria.

**Figure 5:**
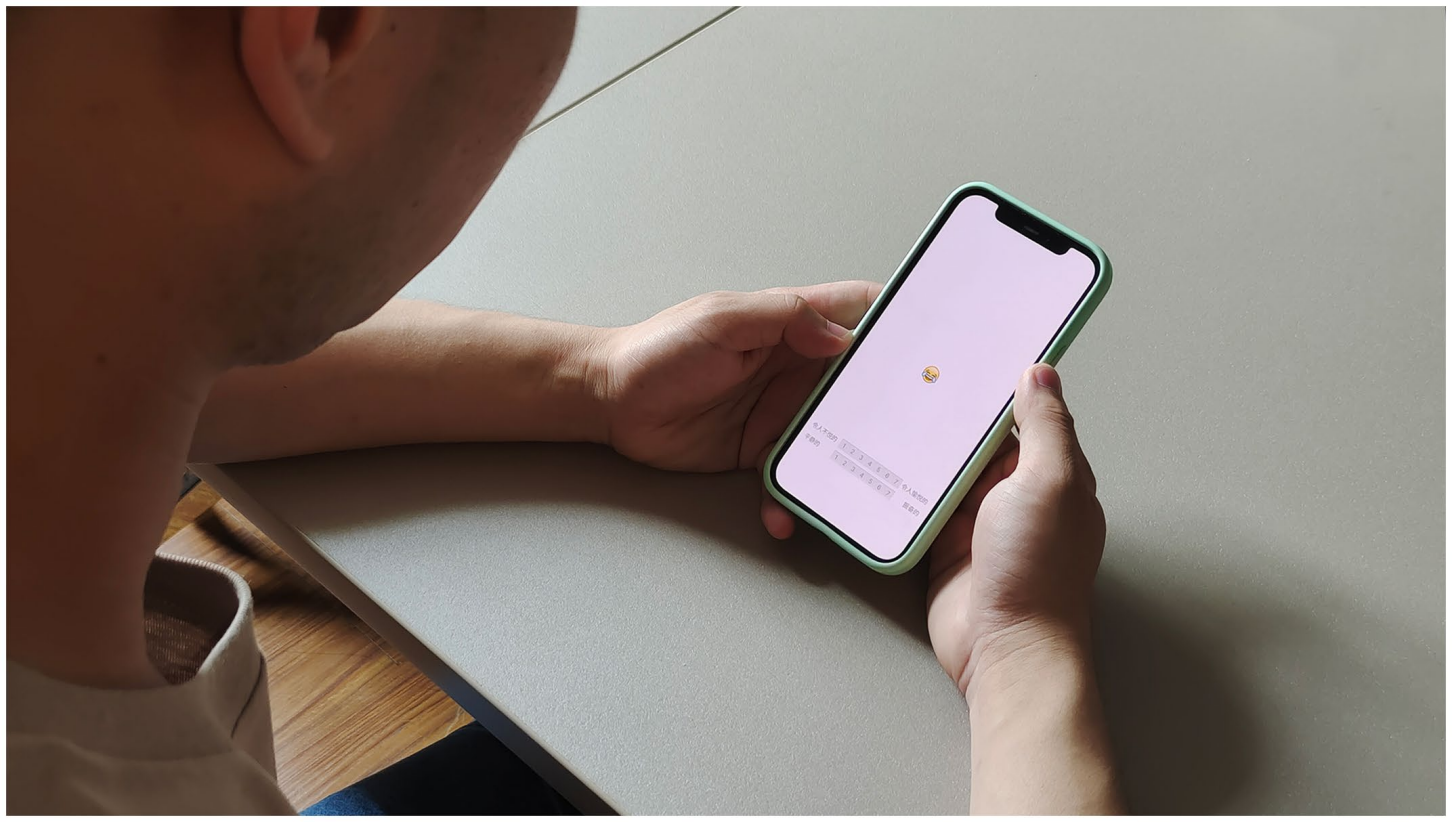
User test scenarios.

The experimenter asked the participants to hold the experimental cell phone in the customary position, as shown in Figure 5, and then click to play the dynamic emoji. Participants told the experimenter their valence and arousal ratings of the current dynamic emojis and could make free comments, then the experimenter recorded the scores and ratings before evaluating the next dynamic emojis. During the experiment, a 1-min break was required for each completed group to avoid perceptual bias caused by fatigue. For each participant, the motion effect order and the rhythm order within the group are random, and the order in which the emojis appear is “Tears of Joy,” “Expressionless,” “Angry,” and “Sleeping.” After rating, participants also conducted a structured interview. The average duration of the experiment for each participant was 50 min.

### 3.6. Data analysis

The data analysis was conducted using IBM SPSS Statistics software. A repeated measures ANOVA was performed to examine the effects on valence and arousal. The factors included motor effect (HS, WIW, TT, SE, S, B, and SN) and rhythm (R1, R2, R4, and R8). The significance of the main effects of motor effects and rhythms was tested, and the significance of the interaction was tested. The Mauchly W sphericity test was carried out, and the results of the one-way ANOVA were used for the data satisfying the spherical hypothesis of *p* > 0.05, and the results of the multivariate test or the correction results of the one-way ANOVA were used for the data of *p* < 0.05 not satisfying the spherical hypothesis. *F*-statistics were validated using Greenhouse–Geisser corrected degrees of freedom, and paired Bonferroni corrected *t*-tests were used for *post hoc* comparisons.

## 4. Results

### 4.1. Rating results

**Figure 6:**
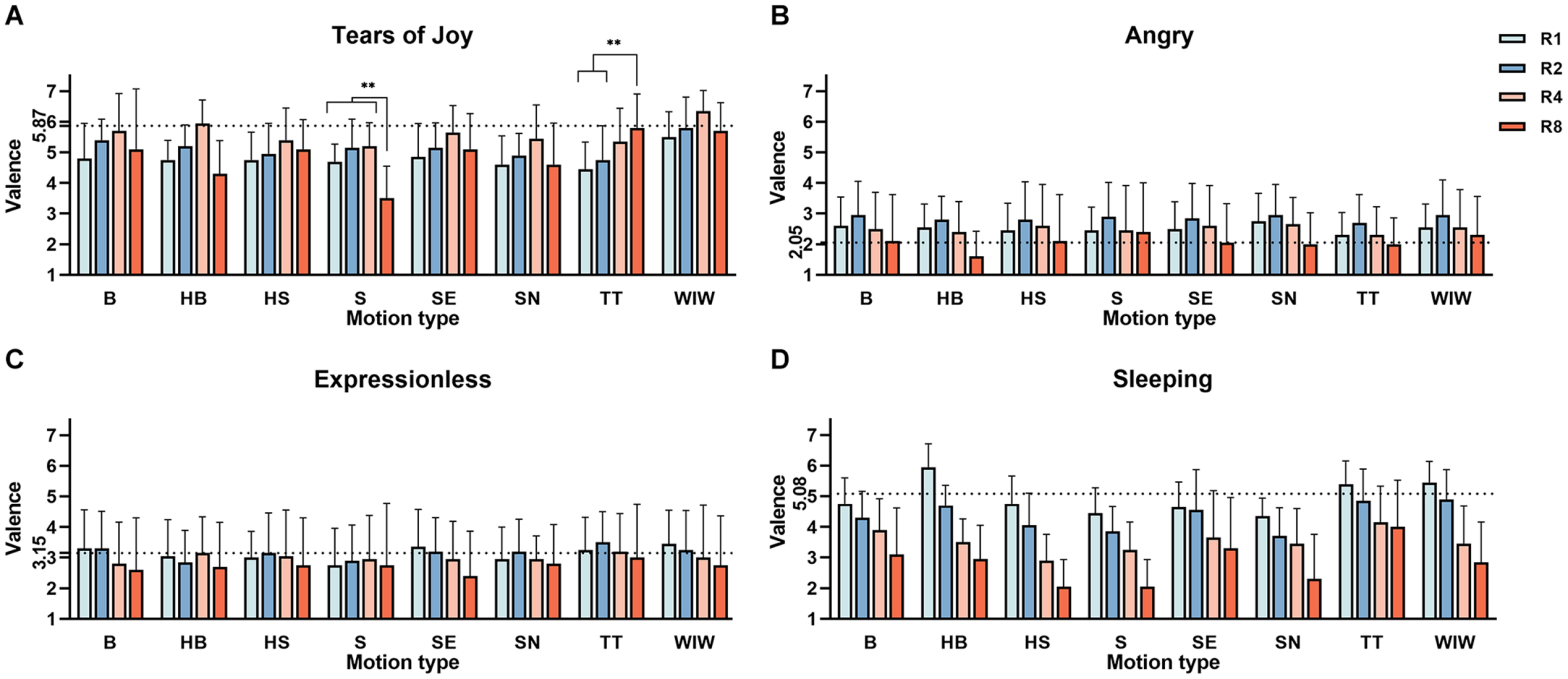
Mean and standard deviation of the valence of different rhythms under different motion effects of four different types of emojis (Mean + SEM). The black dotted line represents the valence rating of this static emoji. Panel **(A)** shows the average valence of 32 different dynamic effects of Tears of Cry. Panel **(B)** shows the average valence of 32 different dynamic effects of Angry. Panel **(C)** shows the average valence of 32 different dynamic effects of expressionless. Panel **(D)** shows the average valence of 32 different dynamic effects of Sleeping. ^**^represents *p* < 0.001, the valence of S-R8 was significantly lower than that of S-R1, S-R2, and S-R4, and the valence of TT-R8 was significantly higher than that of TT-R1 and TT-R2. Only the different rhythms of TT and S showed different valence ratings from the main effect.

**Figure 7:**
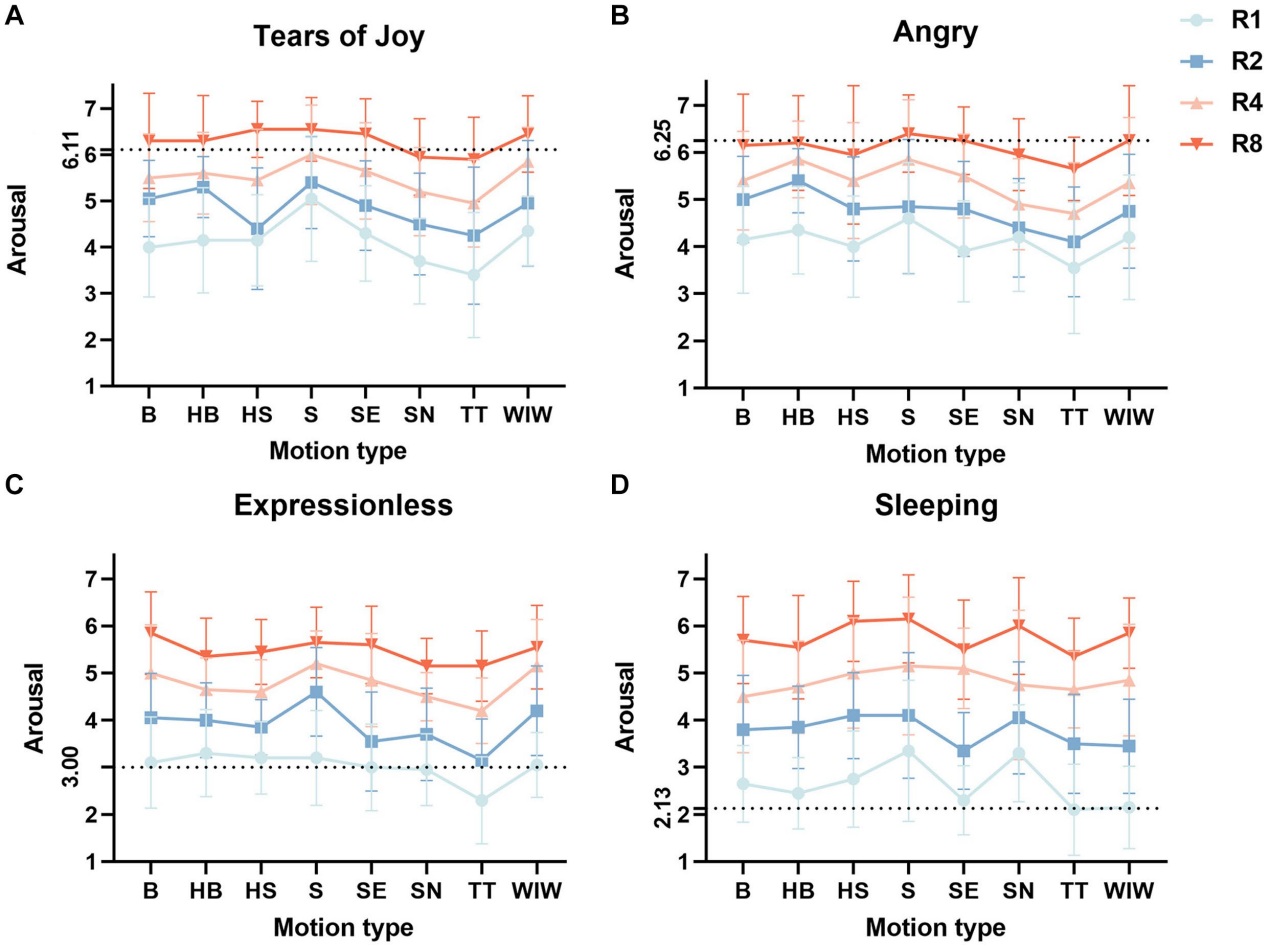
Mean and standard deviation of the arousal of different rhythms under different motion effects of four different types of emojis (Mean + SEM). The black dotted line represents the arousal rating of this static emoji. Panel **(A)** shows the average arousal of 32 different dynamic effects of Tears of Cry. Panel **(B)** shows the average arousal of 32 different dynamic effects of Angry. Panel **(C)** shows the average arousal of 32 different dynamic effects of Expressionless. Panel **(D)** shows the average arousal of 32 different dynamic effects of Sleeping.

The main effect tests and interaction effect tests affecting the valence and arousal of the four emojis are shown in Table 1. The mean and standard deviation of the valence of the four emojis with different rhythms under different motion effects are shown in Figure 6, and the mean and standard deviation of arousal are shown in Figure 7.

**Table 1:** Effects testing of valence and arousal influencing factors.

Emoji type		Tears of joy (HAHV)			Angry (HALV)			Expressionless (LALV)			Sleeping (LAHV)		
		*F*	*p*	η^2^	*F*	*p*	η^2^	*F*	*p*	η^2^	*F*	*p*	η^2^
Valance	Rhythm	29.115	0.000^**^	0.161	22.295	0.000^**^	0.128	7.694	0.000^**^	0.048	181.513	0.000^**^	0.544
	Motion	5.369	0.000^**^	0.198	0.336	0.936	0.015	0.263	0.967	0.012	7.29	0.000^**^	0.251
	Rhythm × Motion	3.816	0.000^**^	0.149	0.462	0.972	0.021	0.674	0.818	0.03	2.859	0.000^**^	0.116
Arousal	Rhythm	210.732	0.000^**^	0.581	170.662	0.000^**^	0.529	341.149	0.000^**^	0.692	402.099	0.000^**^	0.726
	Motion	4.539	0.000^**^	0.173	2.734	0.011^*^	0.112	5.79	0.000^**^	0.21	2.802	0.009^**^	0.114
	Rhythm × Motion	1.129	0.319	0.049	0.93	0.542	0.041	1.403	0.115	0.061	1.492	0.075	0.064

^**^represents *p* < 0.01, ^*^represents *p* < 0.05.

#### 4.1.1. Tears of joy (HAHV)

For valence ratings, rhythms and motion effects showed significant main effects. The *post hoc* comparisons of rhythms showed that R4 had significantly higher valence than R1 (MD = 0.831, 95%-CI [0.60, 1.063], *p* < 0.01), R2 (MD = 0.469, 95%-CI [0.262, 0.676], *p* < 0.01), R8 (MD = 0.731, 95%-CI [0.456, 1.007], *p* < 0.01), and the valence of R2 was also significantly higher than that of R1 (*p* < 0.01), see Figure 6A. The valence of WIW was significantly higher than that of HB (MD = 0.788, 95%-CI [0.118, 1.457], *p* < 0.01), HS (MD = 0.790, 95%-CI [0.128, 1.450], *p* < 0.01), S (MD = 1.200, 95%-CI [0.530, 1.870], *p* < 0.01), SN (MD = 0.950, 95%-CI [0.280, 1.620], *p* < 0.01), and TT (MD = 0.750, 95%-CI [0.080, 1.420], *p* < 0.01).

There was a significant interaction effect between rhythms and motion effects. The effect of partial motion effects on valence scores was statistically significant at four rhythms. The significance results of post-hoc comparisons are shown in Table 2. In R1, the valence of WIW-R1 was significantly higher than that of TT-R1. In R2, the valence of WIW-R2 was significantly higher than that of SN-R2 and TT-R2. In R4, the valence of WIW-R4 was significantly higher than that of S-R4 and TT-R4. In R8, the valence of S-R8 was significantly lower than that of B-R8, HS-R8, SE-R8, TT-R8, and WIW-R8. The valence of TT-R8 and WIW-R8 were also significantly higher than HB-R8. In the same motion effect, the valence of S-R8 was significantly lower than S-R1, S-R2, and S-R4, but the valence of TT-R8 was significantly higher than TT-R1 and TT-R2, which did not conform to the trend of main effects, as shown in the significance labeling in Figure 6A.

**Table 2:** Significance results of *post hoc t*-tests of the valence for motion effects of Tears of Cry at different rhythms.

Rhythm	Motion		MD	SE	*p*	95%CI	
R1	WIW	TT	1.050	0.284	0.008	0.148	1.952
R2	WIW	SN	0.900	0.28	0.044	0.011	1.789
		TT	1.050	0.28	0.007	0.161	1.939
R4	WIW	S	1.150	0.303	0.006	0.186	2.114
		TT	1.000	0.303	0.034	0.036	1.964
R8	TT	HB	1.500	0.393	0.006	0.250	2.750
	WIW	HB	1.400	0.393	0.014	0.150	2.650
	S	B	−1.600	0.393	0.002	−2.85	−0.35
		HS	−1.600	0.393	0.002	−2.85	−0.35
		SE	−1.600	0.393	0.002	−2.85	−0.35
		TT	−2.300	0.393	0.000	−3.55	−1.05
		WIW	−2.200	0.393	0.000	−3.45	−0.95

For arousal ratings, both rhythms and motion effects showed significant main effects, with the post-hoc comparisons of rhythms showing a gradual increase in arousal ratings from R1, R2, R4, and R8 (*p* < 0.01). We found the same trend in the other three emojis, where arousal scores all increased with rhythm (*p* < 0.01), as shown in Figures 7A–D. We will not repeat this point in the subsequent emojis results. The *post hoc* comparisons of motion effects showed that the arousal of S was significantly higher than that of SN (MD = 0.913, 95%-CI [0.180, 1.645], *p* < 0.01) and TT (MD = 1.125, 95%-CI [0.392, 1.858], *p* < 0.01). The arousal of WIW was significantly higher than that of TT (MD = 0.775, 95%-CI [0.042, 1.508], *p* < 0.01). There was no statistically significant interaction effect of rhythms and motion effects on arousal.

#### 4.1.2. Angry (HALV)

For valence ratings, rhythms showed a significant main effect. The *post hoc* comparisons showed that the valence of R2 was significantly higher than R1 (MD = 0.344, 95%-CI [0.119, 0.568], *p* < 0.01), R4 (MD = 0.356, 95%-CI [0.094, 0.618], *p* < 0.01), and R8 (MD = 0.974, 95%-CI [0.513, 1.074], *p* < 0.01). The valence of R8 was significantly lower than that of R1, R2, and R4 (*p* < 0.01). There was a gradual decrease in valence scores from R2 to R4 to R8 (*p* < 0.01), as shown in Figure 6B. The main effect of motion effects was not significant.

For arousal ratings, the *post hoc* comparisons of motion effects showed the arousal of TT was significantly lower than that of S (MD = −0.925, 95%-CI [−1.757, −0.930], *p* < 0.01) and HB (MD = −0.950, 95%-CI [−1.782, −0.118], *p* < 0.01). The interaction effect between rhythms and motion effects on both valence and arousal was not statistically significant.

#### 4.1.3. Expressionless (LALV)

For valence ratings, rhythms showed a significant main effect. The *post hoc* comparisons showed that the valence of R8 was significantly lower than that of R1 (MD = −0.419, 95%-CI [−0.767, −0.071], *p* < 0.01), R2 (MD = −0.450, 95%-CI [0.772, −0.128], *p* < 0.01), R4 (MD = −0.287, 95%-CI [−0.532, −0.430], *p* < 0.01), see Figure 6C. The main effect of motion effects was not significant.

For arousal ratings, the motion effects showed a significant main effect. The *post hoc* comparisons of motion effects showed that the arousal of TT was significantly lower than that of B (MD = −0.800, 95%-CI [−1.356, −0.244], *p* < 0.01), HB (MD = −0.625, 95%-CI [−1.181, −0.069], *p* < 0.01), HS (MD = −0.575, 95%-CI [− 1.131, −0.019], *p* < 0.01), S (MD = −0.962, 95%-CI [−1.518, −0.407], *p* < 0.01), and WIW (MD = −0.787, 95%-CI [−1.343, −0.232], *p* < 0.01). The interaction effect between rhythms and motion effects on both valence and arousal was not statistically significant.

#### 4.1.4. Sleeping (LAHV)

For valence ratings, both rhythms and motion effects showed significant main effects. The *post hoc* comparisons showed that with faster rhythm, the valence rating significantly decreased from R1，R2 (MD = 0.606, 95%-CI [0.381, 0.831], *p* < 0.01), R3 (MD = 1.438, 95%-CI [1.175, 1.700], *p* < 0.01), and R4 (MD = 2.144, 95%-CI [1.831, 2.456], *p* < 0.01), as shown in Figure 6D. There was a significant interaction effect between rhythms and motion effects. Simple effects analysis and multiple comparisons showed statistically significant effects of partial motion effects on valence rating in four rhythms. The significance results of the multiple comparison results are shown in Table 3. In R1, the valence of HB-R1 was significantly higher than that of B-R1, HS-R1, S-R1, SE-R1, and SN-R; the valence of TT-R1 was significantly higher than that of S-R1 and SN-R1; and the valence of WIW-R1 was significantly higher than that of S-R1, SN-R1, and SE-R1. In R2, the valence of HB-R2 was significantly higher than that of SN-R2, and the valence of TT-R2 was significantly higher than that of S-R2 and SN-R2. In R4, The valence of TT-R4 was significantly higher than that of HS-R4. In R8, the valence of TT-R8 was significantly higher than that of HS-R8, S-R8, and SN-R8 (*p* < 0.01).

**Table 3:** Significance results of *post hoc t*-tests of the valence for motion effects of Sleeping at different rhythms.

Rhythm	Motion		MD	SE	*p*	95%CI	
R1	HB	B	1.200	0.246	0.000	0.417	1.983
		HS	1.200	0.246	0.000	0.417	1.983
		S	1.500	0.246	0.000	0.717	2.283
		SE	1.300	0.246	0.000	0.517	2.083
		SN	1.600	0.246	0.000	0.817	2.383
	TT	S	1.950	0.246	0.005	0.167	1.733
		SN	1.050	0.246	0.001	0.267	1.833
	WIW	S	1.000	0.246	0.002	0.217	1.783
		SE	0.800	0.246	0.040	0.017	1.583
		SN	1.100	0.246	0.000	0.317	1.883
R2	HB	SN	1.000	0.307	0.039	0.023	1.977
	TT	S	1.000	0.307	0.039	0.023	1.977
		SN	1.150	0.307	0.007	0.173	2.127
	WIW	S	1.050	0.307	0.023	0.073	2.027
		SN	1.200	0.307	0.004	0.223	2.177
R4	TT	HS	1.250	0.349	0.013	0.14	2.36
R8	TT	HS	1.950	0.418	0	0.621	3.279
		S	1.950	0.418	0	0.621	3.279
		SN	1.700	0.418	0.002	0.371	3.029

For arousal ratings, motion effects showed a significant main effect. The arousal of TT was significantly lower than that of S (MD = −0.788, 95%-CI [−1.528, 0.047], *p* < 0.05). There was a significant interaction effect between rhythms and motion effects. Simple effects analysis and multiple comparisons showed statistically significant effects of partial motion effects on valence rating in four rhythms. The significance results of the multiple comparison results are shown in Table 4. The effect of partial motion effects on arousal ratings was statistically significant in R1. The arousal of S-R1 and SN-R1 was significantly higher than that of TT-R1, WIW-R1, and SE-R1.

**Table 4:** Significance results of *post hoc t*-tests of the arousal for motion effects of Sleeping at different rhythms.

Rhythm	Motion		MD	SE	*p*	95%CI	
R1	S	SE	1.050	0.313	0.028	0.056	2.044
		TT	1.250	0.313	0.003	0.256	2.244
		WIW	1.200	0.313	0.005	0.206	2.194
	SN	SE	1.000	0.313	0.047	0.006	1.994
		TT	1.200	0.313	0.005	0.206	2.194
		WIW	1.150	0.313	0.009	0.156	2.144

### 4.2. Interview results

During the experiment, we collected unstructured feedback from the participants about how they rated the favorability of the emojis and how they felt while rating dynamic emojis. This section will process and analyze these qualitative results.

*Q1: Select your favorite dynamic emoji in each motion effect group and explain the reasons.*

*Q2: Evaluate your feelings about rhythms and motion effects, and you can share your findings and opinions.*

Participants selected their favorite dynamic emoji for each motion effect. Among HS, S, SE, and SN, four participants dropped their choices, resulting in 152 votes. The votes are shown in Table 5.

**Table 5:** Voting status of participants, with slashes indicating that no one voted.

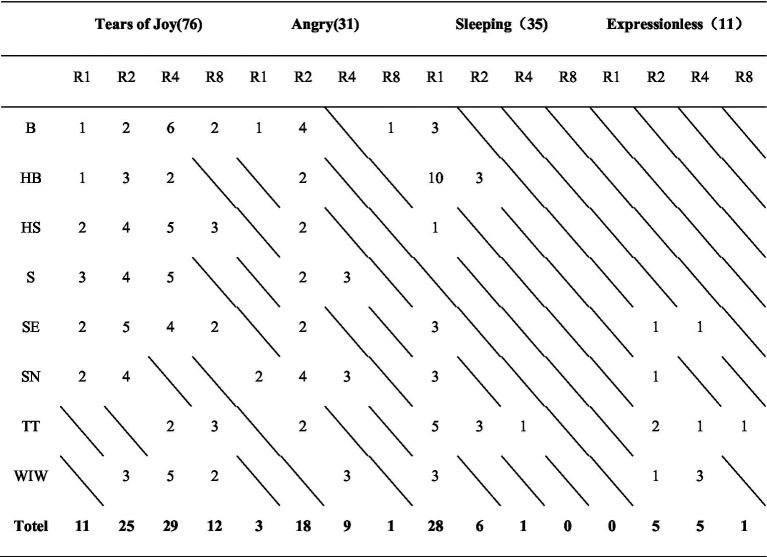

HAHV emojis received the most favorable ratings. “Tears of Joy” (76) > “Sleeping” (35) > “Angry” (31) > “Expressionless” (11). In terms of rhythm type, R4(29) > R2(25) > R8(12) > R1(11) for “Tears of Joy,” R2(18) > R4(9) > R1(3) > R8(1) for “Angry,” R1(28) > R2(6) > R4(1) > R8(0) for “Sleeping,” and R2(5) > R4(5) > R8(1) > R1(0) for “Expressionless.”

Participants also provided feedback on their perception of the dynamic effects. In WIW, half of the participants thought that “Tears of Joy” was joyful and relaxing. Three participants chose “Sleeping” with R1, and P7 (Participant Seven) mentioned that it looked like an old person taking a nap. Among motion effects, HB-R1 was more consistent with the sense of motion of sleeping, and participants thought that the motion effect of “Sleeping” should be slow. P5 thought that “Sleeping” with R1 was very relaxing, much like the breathing of a sleeping baby, and more vivid and immersive than the static emojis. In TT, P3, who selected “Expressionless” with TT-R4, thought that the left-to-right flick looked like an oddity. Those who chose “Sleeping” said it looked like a naughty person swaying with his eyes closed. In HS, four participants mentioned that they felt some oppression, especially when matched with “Angry” and “Sleeping.” They thought that there was a feeling of being hung. In S, most participants reported that they felt some dizziness and could not see the emojis when the rhythm was accelerated. Some other participants thought that this group of motion effects had no clear meaning. In terms of favorability, no one chose high-valence emojis with S either. In SN, nine participants mentioned that they thought the swaying “Angry” could be used to express their true emotions in social situations and that they thought “Angry” with SN-R2 could be a moderate explanation for their “Angry” emotion. Five of the participants expressed their dislike for “Tears of Joy” with SN, mentioning that the left-to-right panning of “Tears of Joy” seemed contradictory and mocking to them. This explained the reason for the low rating of high-valence emojis with SN: the fast-moving, negative-meaning motion effect affected the evaluation of high-valence emojis, resulting in low valence ratings. In B, participants who chose “Tears of Joy” thought the up-and-down bobbing felt like laughing backwards and forwards. In SE, four participants mentioned that SE looked a bit exaggerated. The only two participants who chose “Expressionless,” P6, said that moving “Expressionless” relieved the feeling of silence and made it more interesting, like craning the neck.

## 5. Discussion

In this study, we aimed to explore the emotional reactions to dynamic emojis that were designed based on various rhythms and motion effects. Valence and arousal were used as the measures, and participants were asked to rate 128 dynamic emojis that featured different combinations of rhythms and motion effects. Additionally, participants were also invited to participate in semi-structured interviews at the end of the study to identify their motivations and favorite dynamic emojis.

The results of the study revealed that the impact of rhythms on the arousal level of all dynamic emojis was significant and positive. This suggested that the use of different rhythms in dynamic emojis could effectively enhance the arousal level of individuals. However, the influence of rhythms on the valence level of dynamic emojis varied depending on the specific quadrant. Regarding motion effects, high-valence emojis were significantly influenced by motion effects, but the valence of low-valence emojis was not significant.

Our findings can be divided into the following aspects.

### 5.1. Effects of rhythms on arousal and valence

The effects of rhythms on arousal for all dynamic emojis were significant and positively correlated. As the rhythm accelerated, the arousal ratings consistently and significantly increased. Rhythm is an expression of the time and speed of motion, and the perception of rhythm could be changed by adjusting the interval of motion in animations. Our experiments adjusted rhythm by varying the repetition interval time and speed of different motion effects, and the results showed that emojis with the fastest rhythm (R8) had higher arousal and emojis with the slowest rhythm (R1) had lower arousal. This finding is consistent with the study of online animated advertisements by Sundar and Kalyanaraman (2004), which mentioned that animations with a faster rhythm had higher arousal. We discuss more rhythm types to further confirm the effect of rhythms on arousal.

The effects of rhythms on the valence of all emojis were equally significant, and we also observed different trends in the valence ratings of rhythms for emojis with different emotional types. In the experiment, the valence ratings of LAHV emojis increased as rhythms increased, being most pleasant at the faster rhythm (R4) and starting to decrease at the fastest rhythm (R8). In the study by Kim et al. (2020), the emotion of “vigor” was stronger in short intervals, and “vigor” can be classified as a high-arousal, high-valence form of stimulation. This is similar to our “Tears of Joy.” When short-interval fast rhythm dynamic effects match HAHV emotions, similar to the mechanism of fast rhythm short videos, users secreted more dopamine in a short period of time and felt more pleasure (Meng and Leung, 2021). But the valence rating of the fastest rhythm (R8) became lower, which we believe was related to the speed of the rhythm being too fast. It suggests that the use of interval time should also be controlled within a reasonable range when designing dynamic emojis. In addition, HAHV emojis need to be matched with the faster rhythm’s dynamic effects.

The valence ratings of LAHV emojis decreased as rhythms increased; the valence ratings of the slowest rhythm (R1) were the highest, and the valence ratings of the faster rhythm (R4) were the lowest. In films or cartoons, the rhythm of motion is more rapid for positive emotions such as happiness and victory and generally slower for emotions such as quietness and frustration (Luhta and Roy, 2013). A sense of ambivalence arises when the emotional quadrant in which the emoji itself resides does not align with the emotion conveyed by the rhythm. Aldunate et al. (2018) argued that negative emotions were perceived more strongly for high-valence emojis with inconsistent content and emoji emotions. This is also reflected in the emotional congruence of dynamic effects with emojis. When the accelerated rhythm produced higher arousal, the user felt ambivalent about the low-arousal emojis, which led to unpleasant feelings. Therefore, when matching dynamic effects for LAHV emojis, the slowest rhythm’s dynamic effects should be considered.

Low-arousal and low-valence emojis significantly showed the lowest valence ratings only at the fastest rhythm (R8). The appearance of this result is related to the paradoxical feeling of the fastest rhythm (R8) and low-arousal mentioned above, where the user produces a more unpleasant feeling. “Expressionless” belongs to a neutral category among emojis, but they often have negative meanings (Kralj et al., 2015), and the perceived emotional intensity of neutral emojis was not evident in many experiments (Fischer and Herbert, 2021). Subjects’ emotional perceptions of the LALV emoji varied the most and were closer to the neutral region. We think the first reason is the difference in emotion perception due to cultural differences, and the second is the limitation of the emoji itself, which carries a certain level of arousal because it seems to be a more interesting expression of emojis than just words or images, so it is difficult for the arousal of LALV to fall significantly below a certain level, as reflected in the U-curve (Kutsuzawa et al., 2022). But our results provided at least one point to avoid: Avoid too fast a rhythm for neutral emojis or LALV emojis.

High-arousal and low-valence emojis had the highest valence ratings at the slower rhythm (R2), and the valence ratings decreased as the rhythm became faster (R4 and R8). The faster the rhythm, the higher the arousal becomes. The faster rhythm will amplify the displeasure of low-valence emojis, so users tend to like a slower rhythm’s dynamic effect. However, when combined with the contradictory emotions elicited by the slow rhythm and high-arousal emojis, as well as the user’s evaluation consideration, emojis that are excessively slow may lead to more ambiguity for the user and can also intensitfy the unpleasant feeling. Our results suggest that a slightly slower rhythm’s dynamic effect be designed for HALV emojis.

### 5.2. Effects of motion effects on arousal and valence

For all dynamic emojis, the arousal of TT was significantly lower than that of S. TT had the smallest angle of motion (15 to −15°), while S had a larger angle of motion. Rimé et al. (1985) mentioned that in emotional perception, it was not the shape of the object presented that was important, but the kinetic structure of the motion. This perception is caused by a joint change of speed and direction, so S with a greater change of motion and the fastest rhythm (R8) has a greater sense of visual stimulation for the user and so produces a higher arousal.

In terms of valence, only some of the motion effects had a significant effect on the valence of high-valence emojis but not on the valence of low-valence emojis. Although we did not find a significant association between the motion effect and the low-valence emojis, we determined the valence tendency of the different motion effects in the subsequent user evaluations.

For HAHV emojis, the valence of WIW was the highest in the first three rhythms (R1, R2, and R4). Compared to static emojis, the valence of WIW-the faster rhythm (R4) had the most significant increase in HAHV emojis, which was the more preferred effect in subsequent user evaluations. The valence of S-the fastest rhythm (R8) was significantly lower than that of the first three rhythms for HAHV emojis. Combined with the users’ evaluations, the reason is that after the rhythm is accelerated, fast speed attracts attention (Sundar and Kalyanaraman, 2004), users pay more attention to the way the emojis move, and too fast rhythm makes users unable to recognize the emotional meaning of the emojis themselves, so the decrease in valence is more obvious. In addition, only the valence of TT-the fastest rhythm (R8) was significantly higher. The change in visual stimulation was less pronounced for the smaller motion effect after accelerating the rhythm compared to the other motion effects. We speculate that the reason for this is that the fast rhythm compensates for the visual stimulation caused by the small amplitude of motion, resulting in higher valence ratings. For LAHV emojis, HB-the slowest rhythm (R1) significantly increased the valence of LAHV emojis, and users agree that the effect of it can produce the feeling of sleep and light breathing. WIW-R1 and TT-R1 also produce relatively more pleasurable feelings for the user. In *post hoc* comparisons of the slowest rhythm (R1) and the slower rhythm (R2), the valence of HB, WIW, and TT were higher than that of SN and S. TT also exhibited higher valence than HS in the faster rhythm (R4) and the fastest rhythm (R8). We found that WIW, TT, and HB moved less compared to SN and S. SN carried a negative connotation, and S rotated at too large an angle to give the user a metaphorical feeling that was not positive, so this difference grew as the rhythm accelerated. Users also mentioned that the HS had a sense of suspension, and although the HS moved with the same magnitude as the WIW, the difference in the center point of motion led to a negative feeling for the user.

We found that rhythms and motion effects could modulate high-valence emojis to produce a greater range of valence variation. As shown in Figures 6A,D has a greater range of valence variation, while Figures 6B,C has a smaller range of variation. For low-valence emojis, the rhythms affect their valence ratings at some level but produce a smaller range of valence change.

In general, motion effects can modulate arousal levels to some extent, and motion effects modulate the valence of high-valence emojis significantly but not for low-valence emojis. When matching motion effects to high-valence emojis, it is necessary to consider a combination of motion amplitude and emotions originating from the center of motion.

### 5.3. User favorability and motivation for dynamic emojis

Users’ evaluation of favorability is more based on the valence perception of emojis, and HAHV emojis received the most favorability. In addition, users prefer emotionally connected and metaphorical emojis, while emojis that are unrecognizable or do not conform to dynamics can be confusing to users. Heider and Simmel (1944) found that users attribute different intentions, attitudes, and emotions to the anthropomorphism of moving objects, and that this anthropomorphism can assign human-like emotional and behavioral properties to moving objects. The type of emotion elicited varies according to the motion effects (Rimé et al., 1985). There were also differences in the feelings produced by the eight motion types in our study. Every user who chose HAHV emojis (e.g., “Sleeping”) with HB-R1 mentioned that it looked like a small baby’s breathing and was very quiet. The anthropomorphism generated by emojis was enhanced by the fact that users assigned human-like emotional and behavioral properties to the emojis, and that they were positive emotional metaphors. Similar motion effects also include WIW and TT. S is the motion effect that users associate with the least description because S does not match the realistic way of face motion. It is difficult to generate specific anthropomorphic meanings and situational metaphors, so it is confusing and unpleasant for the user. Additionally, HS has relatively lower likeability, as evidenced by the presence of more negative comments. Some users mentioned that they thought there was a feeling of being hung and that it created a sense of oppression. Furthermore, SN received more negative comments, while B and SE received more neutral comments, indicating a stronger anthropomorphic effect.

Some users also expressed their preference for combining interesting motion effects with low-valence emojis (e.g., “Angry,” “Expressionless”) for some novelty and excitement. For example, in SN and TT, some users also selected low-valence emojis because they perceived “Angry” with SN as a moderate expression of their feelings, and found the side-to-side swaying of the “Expressionless” emoji with TT to be playful. This phenomenon may shed light on the popularity of emojis, many of which exhibit a certain sense of contrast. This sense of contrast enhances the emotional richness for users (Zhou et al., 2017). However, it is worth noting that this point may contradict the earlier discussion on the inconsistency between emojis and dynamic effects. Exploring whether the ambiguity resulting from this contradiction is positive or negative could be a potential direction for future research.

In Figure 7, we can observe the black dotted line which mean the arousal rating of this static emoji. It shows that after adding the dynamic effect, the static emojis with high-arousal had lower arousal, while the emojis with low-arousal had higher arousal. Also, after adding the dynamic effect, the static emojis with high-valence have lower valence, the static emojis with HALV had higher valence, and the static emojis with LALV have lower valence. The addition of dynamic effects seems to have an impact on the valence of high-valence emojis. It is found that dynamics decreased the valence of high-valence static emojis instead of becoming more positive, which is different from the results reported in a previous study (Detenber et al., 1998). The reason is that we were exploring rhythms and motion effects that better matched emojis with different emotion types, so only some of the better matched emojis showed this trend, such as the HAHV emojis of WIW.

### 5.4. Design suggestions

We proposed the following design principles for dynamic emojis:

1. Consider rhythms: emojis with high-valence and high-arousal (HAHV) are better suited to fast rhythm; emojis with high-valence and low-arousal (LAHV) are better suited to slowest rhythm; and emojis with low-valence and high-arousal (HALV) are better suited to slower rhythm; for emojis with low-valence and low-arousal (LALV), avoid using fast rhythm.
2. Consider motion effects: high-valence emojis should match motion effect with more positive metaphorical meanings, otherwise the effect will be sarcastic.
3. Consider the amplitude of motion: consider the match between the amplitude of motion (and direction) and the speed of motion. When the speed of motion is increased, it is advisable to decrease the amplitude of motion to maintain a balanced sense of visual arousal.
4. Consider emotional metaphors: consider kinetic aspects of the metaphor of motion effects to enhance anthropomorphism, which can enhance the user’s perception of emojis.
5. Consider creating contrast: consider matching motion effects or rhythms with contrasts that may make a difference.

## 6. Conclusion

In this study, we introduced dynamic effects of different rhythms and motion effects, matched these dynamic effects with emojis representing different emotions and assessed the emotional responses and liking tendencies of participants. We conducted a user experiment and interviews based on the continuous emotion theory, including valence and arousal. We initially explored how rhythms and motion effects influence arousal and valence, then confirmed the user’s preference and motivation. Finally, we proposed design suggestions for dynamic emojis. These findings were valuable for further designs of dynamic emojis with emotional properties and could significantly contribute to the emotional expression of instant messages in social apps. However, the limitation of this paper is that subjects’ perceptions of LALV emoticons may be influenced more easily by diverse cultural backgrounds, which may affect the scoring of dynamic effects. Therefore, our next research will consider recruiting participants from diverse cultural backgrounds and expanding the four emoji categories to accurately differentiate emoji based on emotional dimensions as well as other dimensions.

## Data availability statement

The raw data supporting the conclusions of this article will be made available by the authors, without undue reservation.

## Ethics statement

The studies involving human participants were reviewed and approved by Sichuan University. The patients/participants provided their written informed consent to participate in this study.

## Author contributions

DY contributed to the drafting, conceptualization, experimental design, and experimental analysis of the manuscript. YR contributed to the related knowledge research and manuscript revision. TY and XD contributed to the preprocessing of the data. MW contributed to the conceptualization and version revision of the manuscript. All authors contributed to the article and approved the submitted version.

## Funding

This study was supported by the Sichuan Provincial Key R&D Project, Grant No. 2021YFG0079-LH.

## Conflict of interest

The authors declare that the research was conducted in the absence of any commercial or financial relationships that could be construed as a potential conflict of interest.

## Publisher’s note

All claims expressed in this article are solely those of the authors and do not necessarily represent those of their affiliated organizations, or those of the publisher, the editors and the reviewers. Any product that may be evaluated in this article, or claim that may be made by its manufacturer, is not guaranteed or endorsed by the publisher.
